# Supplementary material for: Are carbon emissions trading and green financial instruments synergistic? -Comprehensive quantitative research based on content analysis
Source: PLoS One. 2024 Mar 7;19(3):e0298601. doi: 10.1371/journal.pone.0298601 (PMC10919601; doi:10.1371/journal.pone.0298601)
Supplement: S1 Data — (ZIP) [file pone.0298601.s001.zip › Data/地级市Z_F工作报告.zip!/╕ú╜¿/╚¬╓▌╩╨/└┤╫╘╣1⁄2╓┌║┼í╛┬φ┐╦╩2╛▌═°í┐_2016.docx]

各位代表：

　　现在，我代表市人民政府向大会报告政府工作，请各位代表连同《泉州市国民经济和社会发展第十三个五年规划纲要（草案）》一并审议，并请政协委员和其他列席人员提出意见。

　　一、2015年和“十二五”时期经济社会发展回顾

　　2015年，在省委、省政府和市委的坚强领导下，全市上下深入学习贯彻党的十八大和十八届三中、四中、五中全会精神，学习贯彻习近平总书记系列重要讲话精神，紧紧围绕“四个全面”战略布局，着力保稳、求进、攻坚，经济社会保持良好发展态势。

　　（一）突出精准施策，经济保持稳定增长

　　开展“三比一看”活动。进一步提振精气神，有为适应新常态，推动 “比学赶超”。全市生产总值、一般公共预算总收入、规模以上工业增加值、固定资产投资分别增长8.9%、11.3%、8.9%、18.5%。充分发挥政策效应。出台稳增长10条、促进房地产市场平稳发展等综合措施，以及纺织鞋服、建材水暖等5个行业专项政策，开展政策落实评估、督查和整改。全年兑现扶持资金31亿元，争取国家和省级补助124亿元。突出“一企一策”帮扶。坚持每季度开展“帮扶企业发展、促进项目落地”活动周，帮助企业化解困难问题。政府主动站台搞促销，组织品牌“境外行”、“海丝行”和建材“中国行”，支持名优地产工业品开拓市场；推广企业“手拉手”活动，对接产能712亿元。强化投资拉动作用。340个市级在建重点项目完成投资1001亿元；城市公用设施、水利等七大领域新增投资150亿元。39个项目获国家专项建设基金16.1亿元支持。有效防控金融风险。落实政府维护良好金融生态环境的主体责任，政司银保企全线动员，采取“堵、缓、化”方式，协调解决120多家企业资金链问题，为企业提供“过桥”资金541亿元，严厉打击恶意逃废债，金融运行总体平稳。

　　（二）加快转变提升，产业结构持续优化

　　出台进一步推动产业转型升级系列文件，研究制定17个重点产业转型升级路线图。发布“泉州制造2025”发展纲要。实施智能制造、质量品牌、服务型制造3项行动。组织“三个一千”推广计划，兑现智能制造扶持资金近1亿元，建成34个（条）智能工厂、数字化车间和“数控一代”示范生产线，累计引进建设高端科研平台33个。全市新增高新技术企业88家，制修订标准70项，授权专利2万多件。德化获评国家新型工业化产业示范基地。突出培育产业龙头。推动联合石化脱瓶颈、晶安光电（二期）、德润电子等45个制造业重点项目建成投产，实施重点技改项目201个，新增亿元企业105家、中国驰名商标8件，上榜中国品牌价值评价企业20家。强化招商选资，参加省民企对接会和“9·8”投洽会，举办央企和外资（台资）专场招商会，共对接项目277个，总投资2814亿元。中化乙烯、莲花汽车、富德新型能源化工等重特大项目前期工作加快推进。发展特色现代农业。完成农林牧渔业总产值321亿元，粮食播种面积211.6万亩，新增设施农业1.5万亩，新增远洋渔船21艘，新增农民合作社、家庭农场、专业大户等新型农业经营主体1028家，动工建设市区标准化中心粮库。组建农村产权交易平台，新增耕地流转3万亩。深化“第三产业发展年”活动。485个项目完成投资744亿元。出台加快互联网经济发展实施方案，电商交易额超1900亿元、增长35%，快递业务量增长45%，获批中国快递示范城市，德化电商园、安溪弘桥智谷入选国家电商示范基地。中国国际信息技术（福建）产业园建成投用。全市接待游客5125万人次，旅游总收入618亿元，分别增长15.7%和16.9%。出台推进大众创业万众创新措施。重点建设众创空间59个，孵化“双创”企业626家、项目1080个；在全省率先启动网商虚拟产业园建设，入驻企业1700多家。强化人才支撑。深入推进“海纳百川”高端人才集聚计划，新建院士专家工作站4家、博士后科研工作站5家。实施技能人才素质提升工程，新增高技能人才1万多名。出台县域经济特色人才评价办法，评审特色人才500多名。

　　（三）致力先行先试，改革开放步伐加快

　　持续深化金融改革。金融资源加快集聚，恒丰、广发银行泉州分行开业。区域资本市场建设取得新进展，新增境内外上市企业5家、新三板挂牌企业12家，实现直接融资158亿元；海峡股权泉州交易中心挂牌企业410家，海峡金融资产交易中心交易额达180亿元。实体金融成效凸显，全市各项贷款余额5429亿元、比年初增加499亿元，小微企业信贷覆盖率提高到35.1%；中小微企业信用信息交换共享平台导入企业14.8万家，授信280多亿元。对台对外金融合作实现新突破，率先开展泉台跨境人民币贷款业务。深入实施“民综”改革三年行动计划。支持各县（市、区）开展特色主题改革。鼓励引导民间投资，推出PPP项目90个、总投资1050亿元。深化工商登记制度改革，在全省率先开展集群注册，全面实施“三证合一、一照一码”登记，全年新登记市场主体12万家。加快推进“海丝”先行区建设。出台行动方案和发展规划。成功举办第十四届亚洲艺术节暨第二届海上丝绸之路国际艺术节、21世纪海上丝绸之路国际研讨会，“海丝”亚洲艺术公园开园，联合国“海陆丝绸之路城市联盟”工商理事会秘书处落户我市。复制推广自贸区创新举措108项，实现与“海丝”沿线国家和地区进出口额131亿美元。泉州出口加工区升级为综合保税区。泉州晋江国际机场旅客吞吐量达363.6万人次，完成港口吞吐量1.2亿吨、集装箱201万标箱，泉金航线运送旅客12万人次。

　　（四）推进城乡统筹，区域发展更趋协调

　　深化“环湾规划建设年”活动。467个项目完成投资536亿元。开展“多规合一”试点，编制环泉州湾城乡一体化规划，中心城区实现控规全覆盖。深入实施城乡基础设施“六大提升工程”，泉州湾大桥、泉三高速安溪连接线、国道324线丰洛段改造工程等建成通车，湄洲湾南岸铁路支线、厦沙高速泉州段等项目加快推进，泉厦漳城市联盟路泉州段开工建设，刺桐大桥取消收费。进一步规范优化环湾公交线网，投用新能源公交车248辆，着手建设公共自行车租赁系统。出台实施城市交通秩序、卫生保洁、集贸市场、占道经营等四个管理规则，推动文明城市创建机制化常态化。获批国家“智慧城市”试点。加快新型城镇化步伐。晋江“国家新型城镇化综合试点”深入推进，石狮列入“国家中小城市综合改革试点”。21个省市级试点小城镇实施项目641个、完成投资351亿元。有序推进农业转移人口市民化，新办理流动人口居住证77万张。实施宜居环境建设行动计划。新建改造市政提升“五千工程”883公里，建成江滨北路亲水慢道，启动山线绿道工程精品示范段项目，建设人居环境整治村171个、美丽乡村示范（宜居）村32个、美丽乡村景观带9条。拆除改造石结构房屋2914万平方米，拆除“两违” 建筑581万平方米。完成公路安保工程371公里，建设升级农村配电网2038公里。完善农村水利设施，白濑水利枢纽工程项目建议书通过水利部审查，彭村水库主体工程基本完成，泉州向金门供水工程动工建设，农村8.3万人饮水安全问题得到解决。实施精准扶贫，全市减贫5.4万人，造福工程搬迁1.7万人。

　　（五）注重惠民利民，社会事业全面进步

　　全市财政安排民生支出421亿元，占财政支出比重达78%，39件为民办实事项目顺利完成。健全社会保障体系。新增城镇就业15.3万人，农村劳动力转移就业6.2万人。城乡医疗保险、养老保险进一步提标扩面，新增养老床位5980张，新建农村居家养老服务中心（站）100个、农村老年体育活动中心20个，启动机关事业单位养老保险制度改革。加强老龄、老干部和关心下一代工作。不断完善社会救助体系，救助困难群众46万人，惠及低保对象13.4万人，泉港“救急难”工作列入全国综合试点。全市慈善机构增加善款近9亿元。打通商品房与安置房转换通道，开工建设保障性安居工程2.7万套，新增配租配售1.7万套。用好用足住房公积金，全年投放77亿元。落实教育优先发展战略。进一步扩充优质教育资源，新增公办幼儿园学位1万个，新扩容城镇中小学学位2万个。公办普通高中全面取消招收择校生。义务教育阶段外来工子女35.4万人，90%以上在公办学校就读。建成泉州特教学校扩建项目和20个青少年社会教育活动中心。教育信息化建设步伐加快。高等教育、职业教育的人才培养与产业需求更加紧密衔接，建成福大泉港石化学院。提升卫生健康水平。出台深化医药卫生体制改革试点实施方案，启动公立医院综合改革，全面实行药品零差率销售。出台加快推进社会办医的实施意见，全市新增医疗机构床位2000张，市中医联合医院、永春县医院新院等完成整体搬迁，颐和医院奠基建设。加强公共卫生和妇幼保健，有效防控重点传染病。计生服务管理水平进一步提升。推动文化传承发展。出台闽南文化生态保护区总体规划泉州实施方案，扶持建设21个重点区域非遗展示馆、25个传习所、25个重点传承项目，完成17个传统工艺美术濒危艺种的抢救保护任务。惠安、德化分别荣获世界石雕之都、世界陶瓷之都称号。公共文化服务体系不断完善，市四大公共文化中心开工建设。获批国家体育产业联系点城市，圆满完成第一届全国青运会泉州赛区各项工作，成功举办市第十届运动会。加强生态保护治理。通过国家生态市、全国绿化模范城市考核验收。173个重点流域、跨境流域污染整治项目完成投资17.7亿元，第二批小流域“赛水质”活动整治河道55公里，晋江干流水质达标率100%。实现建陶业LNG替代和饰面石材矿山开采全行业退出，完成2005年底前注册营运黄标车淘汰任务，中心市区空气质量优良率达98.9%。开展生猪养殖污染整治攻坚，关闭拆除禁养区生猪养殖场1995家。完成植树造林12.7万亩、水土流失治理24万亩、矿山整治恢复370万平方米。桃溪流域获评国家级湿地公园和水利风景区。深化平安泉州建设。狠抓社会治安综合治理，严厉打击各类刑事犯罪，切实做好信访工作。认真执行市人大进一步加强食品安全监督管理议案的决议，大力治理“餐桌污染”。落实安全生产“党政同责、一岗双责”，加强危化、交通、消防等领域隐患整治，四项指数全面下降。通过全国新一轮双拥模范城考核评估。国防动员、民兵预备役、海防、人防、支前、民族宗教、统计、物价、外事侨务、地震、气象、方志、档案以及青少年、妇女儿童、残疾人等工作取得新成绩。

　　（六）切实转变作风，政府服务提速增效

　　扎实开展“三严三实”专题教育，巩固党的群众路线教育实践活动成果，严格落实“八项规定”，着力解决“四风”问题。认真落实市委全面推进法治泉州建设的实施意见，被赋予地方立法权，政府法制建设得到加强，府院联系机制不断完善，顺利完成“六五”普法规划。自觉接受市人大及其常委会监督，依法执行市人大及其常委会决定决议，主动配合市人大依法履职，办理人大代表建议371件，满意和基本满意率达100%。支持政协履行职能，主动接受市政协民主监督，深化重大事项事前协商，办理政协提案535件，满意和基本满意率达100%。公布实施行政权力清单、公共服务事项清单、责任清单、内资准入负面清单，建立动态调整机制。开展“办事难、办证难”专项整治，推进审批服务提速，复杂事项审批时间压缩至法定时限35%以内。加强廉政建设、行政监察、审计监督等工作，强化效能督查、政务督查，严肃查处“庸、懒、散”行为，加大对失职渎职的追究力度。

　　2015年工作任务的完成，标志着“十二五”规划基本实现。过去五年，我们紧紧把握中央和省里支持泉州发展的重大机遇，深入实施市委“三大战略”，全力推动科学发展跨越发展，有效应对各种风险和挑战，改革开放和现代化建设事业迈上新台阶。

　　这五年，泉州的经济更具规模、更具实力。2015年，全市完成生产总值6138亿元、规模以上工业增加值2903亿元、一般公共预算总收入805亿元、固定资产投资3406亿元，分别比2010年增长71%、104%、101%和182%，经济总量连续17年位居全省首位，人均地区生产总值达7.2万元。形成纺织服装、鞋业、石化、装备制造、建筑建材五大千亿产业集群。高新技术产业增加值达620亿元，占地区生产总值10.1%。

　　这五年，泉州的改革更显深度、更显广度。国家级金融服务实体经济综合改革、民营经济综合改革等获批实施、成效明显。21世纪海上丝绸之路先行区建设列入国家战略。“晋江经验”内涵进一步丰富提升。农业农村、科教文卫、社会治理、政府职能等重点领域改革取得新的突破。2015年全市外贸进出口270亿美元，比2010年增长240%。

　　这五年，泉州的城乡更有品位、更有魅力。连续5年开展“环湾规划建设年”活动，完成投资1900多亿元；环湾建成区面积206.5平方公里，比2010年增加56.5平方公里；全市城镇化率达63.6%，比2010年提高5.2个百分点，海湾型城市框架逐步成型。率先启动美丽乡村建设，实现村庄规划全覆盖，初步建立保洁常态化机制，永春成为全国典型。城乡基础设施更加完善，陆海空交通全面跨越，环城高速闭合成环，实现县县通高速，正式迈入高铁时代、亿吨大港、国际机场行列。

　　这五年，泉州的文化更富美誉度、更富软实力。闽南文化、“海丝”文化的世界影响力进一步彰显，获评中国首个“东亚文化之都”，成功举办2013世界闽南文化节，海上丝绸之路国际艺术节永久落户泉州。全市新增国家文保单位11个、非遗项目6个，新增世界非遗名录1项、记忆遗产1项，成为全国唯一拥有联合国教科文组织全部三大类别非遗名录的城市。

　　这五年，泉州的社会更加和谐、更加文明。全面完成义务教育标准化建设，所有县（市、区）通过国家义务教育基本均衡发展验收。医药卫生体制改革全面推进，新增医疗机构床位1.14万张。城乡医疗保险、养老保险基本全覆盖。人民生活持续改善，城乡居民人均可支配收入分别达37275元和15861元。荣膺全国文明城市，保持国家级科技、卫生、环保、综治、园林等城市荣誉。

　　“十二五”发展成就来之不易，这是省委、省政府和市委正确领导的结果，是全市人民齐心协力、拼搏奋斗的结果。在此，我谨代表市人民政府，向全市广大干部群众，人大代表、政协委员，各民主党派、工商联、无党派人士、各人民团体、离退休老同志，向驻泉省部属单位、驻泉部队、武警官兵，企业界人士和来泉创业务工人员，向广大港澳同胞、台湾同胞、海外侨胞、海内外朋友们，表示衷心的感谢和崇高的敬意！

　　我们也清醒地认识到，泉州正面临应对经济下行和加快转型升级的双重压力：传统产业扩量提质遭遇瓶颈，部分产能外移趋势初显，落后产能转化任务艰巨，三产增速多年低位徘徊；新旧动力还在转换，技术、人才储备不足，大众创业、制度红利等新增长因子作用尚未显现；部分企业生产经营困难，金融风险防控化解任务繁重；区域竞争更趋白热化，高端要素被“虹吸”的压力越来越大；中心城市辐射带动功能不强，农业农村基础仍较薄弱；资源环境约束日益加剧，环境保护压力加大；优质教育、卫生资源相对不足，民生保障与群众期望存在差距；安全生产、食品药品监管、社会稳定还有不少隐患；政府职能转变还不到位，营商环境有待进一步改善。“十二五”规划和2015年部分指标未能达到预期。对于这些问题，我们将高度重视，切实加以解决。

　　二、“十三五”时期主要目标任务

　　未来五年是全面建成小康社会的决胜期，是全面深化改革的攻坚期。对接省“十三五”规划《纲要》，根据市委十一届十次全会通过的“十三五”规划《建议》，市政府编制了“十三五”规划《纲要》（草案）。“十三五”时期我市经济社会发展的总体要求是：全面贯彻党的十八大和十八届三中、四中、五中全会部署，深入落实习近平总书记系列重要讲话精神，坚持“四个全面”战略布局，牢固树立和贯彻落实创新、协调、绿色、开放、共享的发展理念，认真落实中央、省委和省政府支持泉州发展的重大决策部署，以转型升级、跨越发展为主线，以提高发展质量和效益为中心，加快形成引领经济发展新常态的体制机制和发展方式，努力建设创新泉州、智造泉州、海丝泉州、美丽泉州、幸福泉州。

　　“十三五”时期总体目标和重要任务是：到2020年，力争全市生产总值超9000亿元、年均增长8.5%左右，人均地区生产总值超10万元，提前实现地区生产总值和城乡居民人均收入比2010年翻一番。一要打造“创新泉州”。深化创新驱动发展战略，支持企业全方位创新，每个主导产业和新兴产业均形成一个以上高端创新服务平台，力争到2020年全市研发投入占地区生产总值比重达2%，争创国家自主创新示范区。大力推进重点领域和关键环节改革，进一步抓好金融改革、“民综”改革、新型城镇化等国家级试点，形成促进改革创新的体制架构。拓展提升“晋江经验”、“泉州模式”，最大限度激发大众创业、万众创新的活力。二要打造“智造泉州”。着力加强供给侧结构性改革，全面实施“泉州制造2025”，力促主导产业高端化、特色产业集群化、新兴产业规模化，建设“中国制造2025”样板城市。加快现代服务业集聚扩量，着力发展“大旅游、大物流、大健康”产业。培育壮大特色现代农业，促进农村一二三产业融合发展。推动移动互联网、云计算、大数据与传统产业深度融合，提升发展网络经济。力争到2020年，重工业比重达42%、第三产业比重达40%、高新技术产业增加值比重达16%。三要打造“海丝泉州”。围绕建设21世纪海上丝绸之路先行区，全面实施十大行动计划，加快泉州港复兴，办好海上丝绸之路国际艺术节，致力深化与“海丝”沿线国家、地区的政策沟通、设施联通、贸易畅通、资金融通和民心相通，推动泉州与台港澳合作向更广范围、更大规模和更高层次发展。按照全省三大中心城市定位，坚持新城集聚、老城提质，做大做强环湾中心城市，实施古城保护提升计划，建好县域“大城关”及镇域“小城市”。积极稳妥并加快推进新一轮行政区划调整。力争到2020年，环湾区域建成区面积达233平方公里，全市城镇化率达68.5%。大力保护弘扬“海丝”文化，持续建设东亚文化之都和闽南文化生态保护核心区。四要打造“美丽泉州”。实施主体功能区战略，大力推进“多规合一”，加快形成生产空间集约高效、生活空间宜居适度、生态空间山清水秀的国土空间体系。大力发展绿色生产力，因地制宜建设美丽乡村。深入实施大气、水、土壤污染防治，推广绿色循环低碳的生产生活方式，构建生态文明制度体系。五要打造“幸福泉州”。加大民生投入，致力补齐民生短板，推进基本公共服务均等化，健全社会保障体系。大力发展社会事业，加快优质教育、卫生等资源的优化布局和有效供给，培育一批民生领域的知名服务品牌。实施脱贫攻坚工程，推动现行标准下贫困人口全部脱贫。加快构建和谐社会，提高依法治市的能力和水平，加强平安建设，全面提升人民群众的安全感、获得感和幸福感。

　　三、2016年政府主要工作安排

　　2016年是实施“十三五”规划的开局之年，也是推进结构性改革的攻坚之年。经济社会发展主要预期目标为：全市生产总值增长8.5%，农业总产值增长3%以上，工业增加值增长8.5%；一般公共预算总收入增长7%，一般公共预算收入增长5.5%；全社会固定资产投资增长16%；实际利用外资增长6%，出口增长4%；社会消费品零售总额增长11.5%，居民消费价格涨幅控制在3%左右；居民人均可支配收入增长7%左右；完成节能减排任务。

　　为此，我们要在市委统一领导下，坚持稳增长、调结构、强动力、惠民生、防风险，突出供给侧结构性改革，全力推进“五个泉州”建设，确保开好局、起好步。推动经济发展，要注重优化产能、创造增量、攻坚项目、化解风险；推动城市建设，要注重统筹集聚、共建共享、“点线面”提升；推动社会治理，要注重兜住底线、扩量提质、满足多元需求。具体抓好7个方面工作：

　　（一）着力稳中求进，提高经济发展质量效益

　　进一步扩大有效投资。把投资重点放在基础设施、产业升级和民生领域上，突出抓好在建、前期、招商等关键环节。全年实施在建重点项目350个、总投资7390亿元，年度计划投资947亿元。开展“项目前期工作活动年”，强化重大项目协调调度，落实项目建设全过程提速机制，实行“时间倒排、任务倒逼、责任倒追”；结合“帮扶企业发展、促进项目落地”活动周，每季度推动一批重大项目开竣工。加大项目策划、储备和对接力度，每个产业链都打造一支相对稳定、懂业务、能谈判的招商队伍，全力对接一批大型央企、知名民企和重大外资项目。深入实施“回归工程”，大力促成在外乡贤回归投资先进制造业、传统优势产业提升和现代服务业项目。创新投融资体制机制，大力争取国家专项建设基金，通过政银企对接、产业基金、PPP、债券融资等渠道，有效保障项目资金需求。

　　实施产业转型升级路线图。围绕产业发展方向、龙头项目、集聚区域、发展平台、组织架构、政策措施等六要素，进一步找准定位、落实举措，努力在产业链条的关键领域、重点环节取得更大突破。推动制造业高端延伸。实施传统产业转型升级行动，坚持“加减乘除”并举，优化产业生态环境，提升优质资源的集聚和配置能力，真正让主导产业、特色产业“留下来、发展好”，在技术、品牌、市场等方面继续保持国内外领先地位。实施新兴产业倍增计划，重点发展集成电路、新一代信息技术、新材料等产业，力争在中下游环节快速突破、形成规模，加快建设中心市区、安溪、晋江、南安光电信息产业带。突出龙头带动，开工建设中化乙烯、莲花汽车、火炬电子材料、晶安光电（三期）、天电光电等一批扩量提质项目。加快实施“泉州制造2025”，大力推进以“机器换工”为核心的智能化改造，落实新一轮智能装备应用推广计划，新建15个以上示范性智能工厂、数字化车间；乘势培育壮大智能装备产业，建好洛江、晋江、南安等智能装备产业园区。深入开展技术改造，实施重点技改项目200个以上，全市完成技改投资800亿元以上。积极稳妥推进企业兼并重组、化解过剩产能，打造百亿企业、巨型企业；支持中小企业融入产业生态链，围绕大企业开展配套协作，实现优势互补、协调发展。实施质量品牌提升计划，加快创建全国质量强市示范城市，健全检验检测与认证技术服务，鼓励企业参与标准建设，加强知识产权保护，争创国际品牌。加快发展海洋经济，规划建设石狮、台商区、晋江等海洋经济园区。扶持建筑业发展壮大，实施建筑工业化试点。推动现代服务业加快发展。把培育专精服务新业态作为“第三产业发展年”的重要内容。生产性服务业要突出与实体经济有机融合，重点发展电商、物流、工业设计、服务外包等产业，有力支持制造业生产方式、商业模式、供应链服务等转变提升。认真实施“互联网+”行动计划，引导电商平台、专业市场、生产企业无缝对接，推进网商虚拟产业园、“泉州购”综合大型供货平台等项目建设。加快完善物流配套设施，鼓励大数据、云计算在物流领域的运用，抓好菜鸟网络科技、晋江快递产业园等项目。积极发挥新消费引领作用，大力发展健康养老、文体娱乐、休闲养生、家政护理等生活性服务业。加快发展体育产业，发挥我市体育运动品牌优势，举办环泉州湾国际公路自行车赛、南少林五祖拳武术大赛等赛事，促进体育消费。

　　激发创业创新活力。以建设福厦泉国家自主创新示范区泉州片区为契机，推进国家高新区“一区九园”建设，打造新的经济增长极。强化科技公共服务，建好中科院、华中科大、哈工大等在泉高端创新平台，深入千家企业开展技术开发应用和咨询服务。落实推进大众创业万众创新措施，借鉴中关村创业大街运作经验，大力发展众创、众包、众扶、众筹等新模式，整合厂房、设备、金融、技术等要素资源，打造晋江“三创园”等一批具有泉州特色的众创空间。举办众创众筹电视大赛。

　　加强人才集聚服务。实施企业家素质提升培训计划，进一步提高企业家战略把握、形势研判和实操能力。完善柔性引才机制，突出“高精尖缺”导向，加强带团队、带项目、带资金的高层次创业创新人才引进。支持企业与院校开展“订单式”人才培养工作，加快打造一支产业转型升级所需的技能人才队伍。健全人才激励评价体系，探索实行“积分制”，落实人才落户、子女就学等政策，营造良好人才环境。

　　（二）着力夯实基础，扎实推进农业农村工作

　　建设特色现代农业。加强农业供给侧结构性改革，整合设立农业发展专项资金，强化投入精准性，加快推进农业现代化。严格耕地保护，落实粮食储备，加快建设市区标准化中心粮库。实施农业“五新”推广，创建高产示范片35万亩，引进台湾新品种、新技术，推动乌龙茶、芦柑等特色农产品转型升级。推进农业设施化规模化高端化，年度实施项目80个，开工三安植物工厂、道畋农业、喆畋农业等龙头项目。加大农产品品牌建设，加强地理标志产品保护和运作，新增农业“三品一标”认证20个，完善农产品质量安全可追溯体系。突出“接二连三”，加强农产品精深加工，培育发展冷链物流，支持农产品电商平台、农业物联网示范企业建设，大力发展休闲农业、观光农业。推进土地承包经营权有序流转，发展多种形式适度规模经营。积极培育新型农业经营主体，扶持市级农业龙头企业30家、农民合作社示范社30家。发展远洋渔业，规划建设渔港经济区。

　　深入推进新农村建设。注重改善农村人居环境，加强农村自然风光、人文风情、传统风貌建筑保护，抓好220个人居环境整治村、美丽乡村示范（宜居）村和8条美丽乡村景观带建设，让农村“留得住青山绿水，记得住乡愁”。加快完善农村生产生活基础设施，推进县乡公路、农村配电网等提级改造，实施水利项目137个、年度投资55亿元，开工建设白濑水利枢纽工程，全面实施七库连通、山美水库流域生态保护试点等重大项目，抓好水库和海堤除险加固、农村饮水安全等民生水利工程。

　　推进精准扶贫精准脱贫。打好脱贫攻坚战，对接落实赣闽粤原中央苏区振兴发展规划，安排1亿元市级扶贫开发专项资金，广泛动员汇集更多社会扶贫资源，加大对30个扶贫开发工作重点乡镇和300个重点村的支持力度。坚持因人因地施策，做好贫困户全面核查、建档立卡和滚动管理，对丧失劳动能力、缺乏资金技能、因病、因灾、因学等致贫人口实行精准帮扶，推进最低生活保障线和市定贫困线“两线合一”，确保年度脱贫5万人以上。致力增强内生“造血能力”，在贫困地区实施一批基础设施、生态建设和民生工程项目。推进易地扶贫搬迁，完成省市搬迁任务。支持薄弱村发展村集体经济。

　　（三）着力重点攻坚，深化关键领域改革开放

　　推进体制机制创新。深化金融改革。致力拓展金融资本进入实体经济、民间资本进入金融领域“两个通道”，推动小微企业信贷覆盖率37%以上，直接融资占社会融资比重达14.5%。建设金融业态集中区，争取获批民营银行、金融租赁公司，做大做强泉州银行，推动准金融机构创新发展。优化实体金融服务，深化全产业链金融服务试点，完善中小微企业信用信息交换共享平台功能，扩大小微企业贷款保证保险业务规模，提升农村普惠金融服务覆盖率和可获得性。加快发展区域资本市场，累计实现上市企业100家以上、“新三板”挂牌企业25家以上、海峡股权泉州交易中心挂牌企业500家以上。深化对台对外金融合作，扩大泉台跨境人民币贷款规模，加快推进外资企业资本金意愿结汇。维护良好金融生态，完善守信激励和失信惩戒机制，规范发展民间金融，开展打击恶意逃废债专项行动，坚守不发生系统性、区域性金融风险底线。深化“民综”改革。开展降低实体经济企业成本行动，进一步激发民营经济活力。深入开展“一照一码”改革，加快推进全程电子化登记和电子营业执照应用，提升注册便利化水平。发展壮大产业股权投资基金，加快设立新兴产业、高新技术、集成电路、重整并购、城市发展等专项子基金，鼓励民间资本参与。积极拓展公共资源市场化配置范围，整合市、县公共资源交易平台。深化资本、资产等领域改革。抓好农村宅基地和集体建设用地使用权确权登记颁证，推进晋江国家级农村宅基地制度改革试点。全面开展土地承包经营权确权登记颁证，稳妥实施土地承包经营权和农民住房财产权抵押贷款试点。启动不动产统一登记工作。抓好国资、供销社、林业、价格等方面改革，推动国有企业重组整合、发展壮大。

　　推进高水平双向开放。加快“海丝”先行区开放合作。认真落实行动方案和发展规划，重点抓好中兴海丝路跨境电商服务平台、海丝指尖物流公共服务平台等97个早期收获项目。积极推动 “海丝”沿线国家和地区来泉共建石油化工、汽车制造等产业合作园区。办好第二届“海丝”国际品牌博览会。加强与国际友城的合作。提高投资贸易水平。坚持“请进来”与“走出去”双联动。创新利用外资方式和工作机制，加快培育利用外资新增长点。落实外贸扶持政策，打造服务企业“走出去”的政策、金融、风险预警等综合保障平台；大力支持企业赴境外办展参展，抱团开展国际产能合作、品牌并购、资源开发和营销网络布局。主动复制推广自贸区制度创新经验，加快实行“单一窗口”和“三互”大通关。促进泉台融合发展。高水平推进台商投资区开发建设。积极组织赴长三角、珠三角等台资密集区开展招商，有针对性引进台湾微电子、光电、石化等产业链关键项目，抓好台湾海峡两岸医事交流协会系列项目落实。支持台资企业转型升级。致力增进泉台人文情感交流，拓展直接往来，促进客货运直航发展壮大。完成泉州向金门供水工程建设。深化泉港澳侨合作。落实与香港合作三年行动方案，加快南洋华裔族群寻根谒祖平台、华侨捐赠公益事业资料库等项目建设，做好新华侨华人和华裔新生代工作。

　　（四）着力统筹集聚，做大做强环湾中心城市

　　推进城市“点、线、面”提升。在城市总体规划和环湾向湾发展的框架下，强化城市设计，探索“低冲击”开发模式，从城市的关键节点、组团连线、重点片区入手，推动规划从墙上走到地上，加快塑造城市崭新形象。“点”，要突出抓好重大城建项目、标志性工程，组织实施城建重点项目395个，年度计划投资超500亿元，打造市公共文化中心、东海会展中心、泉州歌舞剧院、泉州植物园等城市精品点。“线”，要突出完善城市山体系统、滨水系统和快速路网，制定实施“两江”沿岸景观提升规划，抓好晋江下游生态整治工程、山线绿道、崇武至秀涂海岸带等城市风景线，提高山江湖海的易达性，让山水走近城市，让人们亲近自然。“面”，要突出重点区域城市空间轮廓线控制，统筹建筑布局，协调景观风貌，围绕东海滨海片区、百崎湖周边等区域，完善开发策略、业态布局、项目支撑，推动要素集聚、功能配套、产城融合，培育城市高档商圈、总部经济、众创空间等现代服务业集聚区，打造若干示范片区。修订出台“三旧”改造政策，引导旧厂房改造发展新兴业态。有效化解房地产库存，完善“泉房通”商品房与保障房购销服务平台，加快棚户区和石结构房屋改造，力争征迁货币化安置比例不低于50%。抓好新型城镇化各级各类试点。

　　强化基础设施支撑。加快环湾路网建设，推进重要主干道和交通节点改造提升，开工建设现代有轨电车、城东至北峰快速通道，提高跨组团快速通达能力。优化对外互联互通，开工建设兴泉铁路、福厦客专（高铁）、泉州综合交通枢纽，加快泉厦漳城市联盟路（高速）、厦沙高速泉州段等建设步伐。突出以港兴城、港城联动，修订完成《泉州港总体规划》，出台港口发展三年行动计划，抓好石湖5#-6#泊位、湄洲湾航道（三期）等项目，科学论证建设秀涂人工岛，开展泉州湾国际邮轮码头规划，完善港口集疏运体系和港后区配套，扶持集装箱运输发展，培育壮大港口经济。实施新一轮宜居环境行动计划，动建垃圾处理产业园，抓好省级“海绵城市”试点，建设新区地下综合管廊示范工程，加强中心市区防洪排涝、内沟河截污工作。

　　创新城市治理方式。完善市县两级规划管理体制，强化“多规合一”，制定规划建设“负面清单”，加强对古城、新区、“两江”、南北翼的规划统筹和有效管控。有序推进农民工市民化，提高户籍人口城镇化率。加快“智慧城市”建设，抓好智能交通、智慧医疗、智慧社区等信息惠民工作，建设数据交换共享平台，完善城市应急体系。实施城市畅通工程，优先发展公共交通，加快公共自行车租赁系统运行，抓好公共停车场建设，做好城市道路微循环系统规划和改造。深化城市执法体制改革，加强城乡“两违”源头监管和分类治理。抓好新型社区治理试点，完善物业协调管理体系。落实创建全国文明城市常态化机制，培育志愿服务文化，鼓励市民参与城市治理，实现共治共管、共建共享。

　　（五）着力传承弘扬，深度挖掘释放文化的力量

　　擦亮千年古城名片。增强历史文化名城保护意识，把古城作为泉州的最大财富、最亮名片进行策划提升。坚持修旧如旧，完成历史文化名城保护规划修编，注重街区整体提升与个体建筑修缮相结合，抓好西街、中山路保护整治及闽南文化生态园等项目，保持古城格局和风貌的完整性。探索建立“千年古城、家园共造”的管理机制，提升古城居住环境和服务能力，推动生活方式、风俗习惯活态传承，精心培育发展适合业态，让古城“见人见物见生活”。加强对历史名街、名镇、名村、名人故居等保护整治。

　　集中打响文化品牌。主动担起传承弘扬“海丝”文化的责任，大力推进“海丝”申报世界文化遗产工作，加强“海丝”遗产点修缮保护和环境整治，建设中国海上丝绸之路博物馆。弘扬闽南文化，深化提升“十百千”基础工程，建好29个“非遗”生产性保护示范基地，继续抓好21个闽南文化整体性保护重点区域建设。加强文物保护。扶持泉州戏曲传承与发展。

　　大力发展文化旅游。实施文化产业倍增计划，抓好源和1916、领SHOW天地等建设，鼓励发展文化新业态，促进大众文化消费。创新发展“1+N”大旅游模式，推动旅游与文化、宗教、工业、农业、休闲等深度融合。加强“海丝泉州”、“东亚文化之都”旅游品牌宣传推广，策划推出一批特色旅游主题和精品线路。推进泉州古城旅游、安永德山区绿色生态旅游、崇武和永宁滨海旅游等项目，加快A级景区、旅游特色街区、旅游集散中心等建设，引进和培育发展知名旅行社。

　　提升公共文化服务。深入实施“万千百十”文化惠民工程，抓好少儿图书馆、城市街区24小时自助图书馆等项目，推进基层文化设施达标提升，提高公共文化数字化水平。加快广播电视节目无线数字化覆盖，积极推动文艺演出及文艺精品创作创新。推进第二轮修志工作。办好第八届亚洲世界语大会。

　　（六）着力绿色发展，建设国家生态文明示范市

　　铁腕重拳治理环境污染。全面落实水污染防治行动计划，严格实行“河长制”，加强饮用水源保护，突出生活污染、工业污染、畜禽养殖污染专项治理，深化重点流域、跨境流域整治和小流域“赛水质”活动，建设安全生态水系。认真落实大气污染防治行动计划，重点整治工业粉尘、餐饮油烟、建筑施工、垃圾焚烧等污染源，改善空气质量。制定实施土壤污染防治行动计划，开展耕地重金属、农田残膜污染治理，加快固废、危废处置中心建设。严厉打击各类环境违法行为。

　　推进资源节约集约利用。深化能效对标行动，充分挖掘企业节能潜力，实施节能重点项目50个以上，抓好燃煤锅炉节能环保提升工程。深入推进德化陶瓷园区、泉港石化园区循环化改造，开展石狮国家级循环经济示范市创建工作。继续实施最严格水资源管理制度，争创国家节水型城市。推进节约集约用地，盘活利用闲置土地，建设绿色矿山。发展农村沼气工程。

　　完善生态文明制度体系。严格落实生态保护红线管控措施，完善森林生态效益和“两江”流域生态保护补偿机制。强化重点生态功能区和生物多样性保护，加强山体、水域、湿地、海岸带生态修复，抓好造林绿化、水土保持，创建国家森林城市。推进重点生态区位商品林赎买试点。推广实施环境污染责任保险，探索推行环境污染第三方治理。实行生态环境损害责任终身追究制。

　　（七）着力补齐短板，不断提高人民幸福指数

　　织牢就业和社会保障安全网。落实就业创业扶持政策，设立创业担保贷款专项资金，统筹抓好高校毕业生、就业困难人员等重点群体就业。开展“全民参保登记计划”试点，进一步提高社保覆盖面，实行城乡居民基本医疗保险政策一体化。加快金保工程建设，实现社保数据跨部门、跨区域交换共享。构筑完善养老服务体系，抓好市社会福利中心等项目，建设居家养老服务信息平台，推进医疗卫生和养老服务融合发展。深化机关事业单位养老保险制度改革。强化兜底责任，提高城乡居民最低生活保障水平，深化“救急难”试点，建立健全救助标准自然增长机制。发展慈善公益事业和残疾人事业。深入实施妇女儿童发展纲要。关心关爱农村“三留守”群体。

　　提高教育发展水平。基础教育要突出增加优质资源、推动均衡发展，实施新一轮学前教育三年行动计划和特教三年提升计划，新改扩建公办幼儿园15所，实现特教学校标准化建设；发挥“名校、名师、名校长”辐射带动作用，加快城东、东海、江南学园和台商区实验学园等项目建设，推进农村义务教育薄弱学校改造和城镇中小学扩容，全市新增学位2万个。高等教育、职业教育要突出服务产业转型升级，推行现代学徒制，建设一批示范性现代职业院校、职业教育集团和生产性实训基地；启动职业院校专业群对接产业群建设工程，打造有竞争力的特色学科和专业。扶持和规范发展民办教育。抓好老年教育、终身教育。持续加强和改进未成年人思想道德建设，开展中小学劳动教育实验，做好关心下一代工作。

　　加快卫生计生事业发展。医药卫生体制改革要以解决看病难、看病贵为重点，突出“三医”联动，扩充优质卫生资源，努力满足人民群众多层次、多样化的健康需求。新建市妇产医院、老年医院、颐和医院，扩建一院新院、福医大附属二院新院、医高专附属人民医院、光前医院，续建石狮医院新院、晋江医院新院和泉州滨海医院，当年全市新增医疗机构床位2000张以上。深化公立医院综合改革，建立现代医院管理制度，加强县级医院综合能力和基层医疗服务体系建设，加快培养引进紧缺急需卫生人才。大力推行分级诊疗，建立健全分工协作、双向转诊、财政补偿制度。落实基本公共卫生服务，推进妇幼保健和计生技术服务资源整合共享。加强传染病和慢性病防控。积极创建全国基层中医药先进市。落实“全面两孩”政策，发挥计生协会作用，加强基层计生工作。深化全民健身运动，倡导健康生活方式。

　　进一步强化社会治理。完善矛盾纠纷多元化解机制，推进“阳光信访、责任信访、法治信访”。深化社会治安综合治理，依法严厉打击违法犯罪活动。建立健全食品安全社会共治体系，严厉整治食品药品违法行为。抓好道路交通、消防、危化、建筑施工等重点行业领域安全生产，加快泉港石化园区安全控制区建设，争创全国安全发展示范城市。支持工会、共青团、妇联等人民团体发挥更大作用。深入开展民族团结进步创建活动。加强宗教事务依法管理。持续推进军民融合深度发展，实施一批民参军、军转民重点项目，打造双拥建设升级版。

　　四、切实加强政府自身建设

　　各位代表！发展新起点，人民新期待，要求我们必须全力推进政府建设，加快职能转变，以更加开阔的视野、更加昂扬的斗志、更加务实的作风，推动泉州新一轮跨越发展。

　　始终坚持解放思想、创新争先。要爱拼敢赢、争创一流，高标准、高要求推动“十三五”发展，保持全省平均水平之上，确保“红旗不倒”。要创新理念、创新方法，坚决破除各种思维定势和体制障碍，推出一批具有重大牵引作用的改革举措，进一步开拓泉州发展新境界。要主动担当、敢于负责，不满足于一般化，勇于突破发展中遇到的复杂问题和深层次矛盾，在各个岗位上创造新业绩，展示新形象，体现新作为。

　　始终坚持执政为民、人民至上。时刻不忘政府前面“人民”二字，始终把人民利益放在第一位，努力把市民的期待变成我们的行动，把市民的希望变成生活的现实。坚持保基本、兜底线、补短板，把新增财力更多用于民生，抓好为民办实事项目，不断加大公共服务产品供给。巩固“三严三实”专题教育成果，弘扬“滴水穿石”、“四下基层”的好作风，严肃查处损害群众利益的行为。积极开展接访、走访、回访活动，真诚倾听群众呼声，真实反映群众愿望，真情关心群众疾苦。

　　始终坚持简政放权、务实高效。经济形势越是严峻，越需要政府主动作为、靠前服务。要对标国内国际先进，从影响企业开办、营运、退出等10个领域入手，建立与国际接轨的营商规则和评价体系。落实清单管理模式，优化审批流程，清理规范中介机构。深化“互联网+”政务服务，打造“指尖上的行政服务中心”。强化效能督查，杜绝“吃拿卡要”，有效治理慢作为、不作为、乱作为。充分发扬政企互动的好传统，旗帜鲜明支持企业经营发展，理直气壮维护企业合法权益，不遗余力为企业排忧解难，进一步夯实泉州实体经济的根基。

　　始终坚持依法行政、清正廉洁。认真贯彻《中国共产党廉洁自律准则》和《中国共产党纪律处分条例》，全面落实党风廉政建设主体责任和监督责任。自觉接受人大法律监督、工作监督和政协民主监督，提高人大代表建议和政协委员提案的办理质量，充分听取各民主党派、工商联、无党派人士以及各人民团体的意见和建议，广泛接受新闻舆论和人民群众监督。加强行政监察和审计监督。抓好公务用车制度改革。强化法治政府建设，做好地方立法工作，全面启动“七五”普法，深化政务公开，加强行政复议和行政应诉工作，拓展府院良性互动内涵，进一步提升法治泉州建设水平。

　　各位代表！泉州人有精神，企业有根，社会有活力，泉州的发展前景无限广阔！让我们紧密团结在以习近平同志为总书记的党中央周围，在中共泉州市委领导下，紧紧依靠全市人民，脚踏实地，锐意进取，为建设“创新、智造、海丝、美丽、幸福”的现代化泉州，为在更高水平上全面建成小康社会而努力奋斗！
